# Identification of Internal Defects in Potato Using Spectroscopy and Computational Intelligence Based on Majority Voting Techniques

**DOI:** 10.3390/foods10050982

**Published:** 2021-04-30

**Authors:** Kamal Imanian, Razieh Pourdarbani, Sajad Sabzi, Ginés García-Mateos, Juan Ignacio Arribas, José Miguel Molina-Martínez

**Affiliations:** 1 Department of Biosystems Engineering, College of Agriculture, University of Mohaghegh Ardabili, Ardabil 56199-11367, Iran; k.imanian@uma.ac.ir (K.I.); s.sabzi@uma.ac.ir (S.S.); 2 Computer Science and Systems Department, University of Murcia, 30100 Murcia, Spain; ginesgm@um.es; 3 Department of Electrical Engineering, University of Valladolid, 47011 Valladolid, Spain; jarribas@tel.uva.es; 4 Castilla-Leon Neuroscience Institute, University of Salamanca, 37007 Salamanca, Spain; 5 Food Engineering and Agricultural Equipment Department, Technical University of Cartagena, 30203 Cartagena, Spain

**Keywords:** potato, spectroscopy, internal defect, majority voting

## Abstract

Potatoes are one of the most demanded products due to their richness in nutrients. However, the lack of attention to external and, especially, internal defects greatly reduces its marketability and makes it prone to a variety of diseases. The present study aims to identify healthy-looking potatoes but with internal defects. A visible (Vis), near-infrared (NIR), and short-wavelength infrared (SWIR) spectrometer was used to capture spectral data from the samples. Using a hybrid of artificial neural networks (ANN) and the cultural algorithm (CA), the wavelengths of 861, 883, and 998 nm in Vis/NIR region, and 1539, 1858, and 1896 nm in the SWIR region were selected as optimal. Then, the samples were classified into either healthy or defective class using an ensemble method consisting of four classifiers, namely hybrid ANN and imperialist competitive algorithm (ANN-ICA), hybrid ANN and harmony search algorithm (ANN-HS), linear discriminant analysis (LDA), and k-nearest neighbors (KNN), combined with the majority voting (MV) rule. The performance of the classifier was assessed using only the selected wavelengths and using all the spectral data. The total correct classification rates using all the spectral data were 96.3% and 86.1% in SWIR and Vis/NIR ranges, respectively, and using the optimal wavelengths 94.1% and 83.4% in SWIR and Vis/NIR, respectively. The statistical tests revealed that there are no significant differences between these datasets. Interestingly, the best results were obtained using only LDA, achieving 97.7% accuracy for the selected wavelengths in the SWIR spectral range.

## 1. Introduction

Potatoes, as one of the most important agricultural products in the world, play an important role in providing food. Since potato is nutrient-rich, it can be easily attacked by pests and diseases [1]. Potatoes are susceptible to various diseases, some of which are widespread, and others have a limited diffusion and are local. The origins of these infectious diseases include bacteria, fungi, viruses, mycoplasmas, viroids, and nematodes [2,3]. Another group called physiological, non-infectious, diseases include complications due to adverse weather conditions, nutrient deficiencies, or other non-living factors [4,5]. The applications of common methods for diagnosing some of these internal diseases is destructive, difficult, or even impossible, because the diseases do not have any visible symptoms. Early detection of defects and diseases, in order to separate the products before storage leads to the prevention of disease transmission and increased marketability [6].

The study area of the present paper, in the West of Iran, is one of the most susceptible areas for potato production due to its temperate climate. However, its export value is decreasing due to the decline in product quality owing to several factors: lack of access to healthy tubers for cultivation; lack of crop rotation leading to increasing pests, diseases, and weeds; and lack of storage with favorable conditions, etc. Most of the observed defects are not externally visible, such as brown rot, hollow heart, black heart and flesh discoloration.

Non-destructive analysis experiments use methods without destructive effects of photo-physical, thermal, chemical, mechanical, and photochemical nature [7,8]. Numerous methods have been developed so far to assess the quality of agricultural products, but only some of them have been able to meet the demand and were technically and industrially justified. Among these non-destructive methods, magnetic resonance imaging (MRI) has the highest accuracy, but one of its major disadvantages is the impact of measurement speed on the accuracy, in addition to its high cost. Moreover, it is not recommended for fruits with low moisture content [9]. The application of x-rays in the online inspection of agricultural products has been reported, since this method is sensitive to the mass density of the substance, not to chemical compounds [10]. Ultrasonic waves are also used for measuring the quality of crops, but its development faces serious challenges because it requires measuring the ultrasonic properties of different agricultural products.

Among the non-destructive methods for quality control of products, machine vision and spectroscopy techniques have a promising prospect in agricultural science. These technologies are used in various fields such as analysis of ground and aerial mapping of natural resources, crop monitoring, precision agriculture, robotics, automated guidance, non-destructive inspection of products, quality control, predicting the chemical properties of products, and so on [11]. Therefore, they can be used to quickly determine the quality of agricultural products both on a laboratory scale and in online processing [12,13,14].

Phytochemical, morphological, and physiological processes can lead to changes in the natural spectral behavior of the plants [15,16]. For example, Haase [17] investigated raw potatoes using near-infrared spectroscopy at the range of 850–2500 nm. The properties of dry matter, sugar, and starch were predicted. The results revealed that coefficients of determination, R^2^, were about 0.99, 0.66, and 0.96 for dry matter, sugar, and starch, respectively. Zhou et al. [18] assessed the possibility of classifying potatoes with blackheart using partial least squares-linear discriminant analysis (PLS-LDA) and visible (Vis)/near-infrared (NIR) spectroscopy at the range of 513–850 nm. According to the analysis, wavelengths of 698, 711, 741, 817, and 839 nm were determined as the most effective for the identification of potatoes with blackheart; the total correct classification rate obtained was 96.82%. Moslemkhani et al. [19] used a spectroscopy technique to detect tomatoes infected by the Y virus (PVY) at the range of visible and a part of NIR. They concluded that wavelengths of 900–1100 nm were strongly sensitive to the PVY infection. The linear discriminant analysis was modeled, and the results showed a suitable potential to detect virus-infected plants. Escuredo et al. [20] studied the physicochemical properties of potatoes, including their soluble solid content (SSC), dry matter, phenols, antioxidant, texture, and color features at L*a*b* color space using NIR spectrum. A strong relationship between the color features and the antioxidant components was identified by Spearman correlations. Modified partial least squares (MPLS) regression and principal component analysis (PCA) were used to model the best equations for predicting the mentioned properties. Their results indicated that NIR technology was able to rapidly predict the quality parameters of potatoes. Sanchez et al. [21] conducted a review on assessing the quality of raw and sweet potato using imaging and spectroscopy. They concluded that spectroscopic techniques were more reliable and economical than conventional analytical methods. Moreover, according to the reviewed research, potato and sweet potato were physiologically similar; therefore, challenges should be faced for online classification of sweet potato, and it is necessary to develop advanced non-invasive techniques since the quality of food is considered highly more important than the cost. Marino et al. [22] classified potatoes based on external defects, including black dot, damaged, black scurf, greening, and common scab using a supervised learning method. A new labeled dataset was created. Then, a convolutional neural network (CNN) was trained to conduct the classification task based on coarse-to-fine segmentation. The results revealed that CNN was able to classify potatoes with a recall of 0.90 and precision of 0.91.

According to previous research, it is found that many studies have been accomplished on potatoes, most of them focused on the detection of visible defects and normally using a single detection algorithm. The innovation of the present study is the proposal, development, and validation of an ensemble classifier, combined with the majority voting rule [23] that includes hybrid artificial neural networks (ANN) and imperialist competitive algorithm (ANN-ICA), hybrid ANN and harmony search algorithm (ANN-HS), linear discriminant analysis (LDA), and k-nearest neighbors algorithm (KNN), to identify internal defects of potatoes that have no visible symptoms.

## 2. Materials and Methods

**Figure 1:**
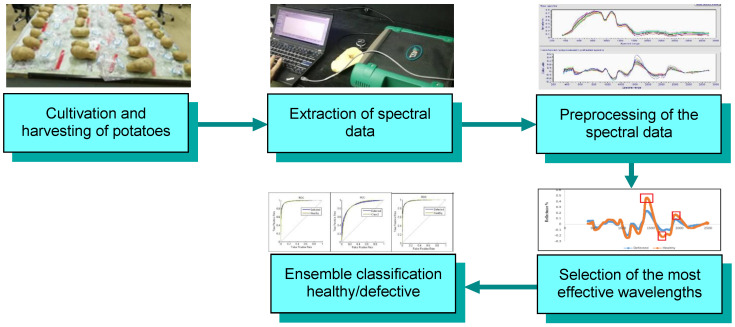
Stages of the applied methodology for detecting deficiencies in potatoes using Vis/NIR spectroscopy.

The research process followed in this research consisted of five main steps, from the cultivation and collection of the samples to the classification using the majority voting method, as presented in Figure 1. These stages are described in the following sections.

### 2.1. Potato Preparation in the Farm

**Figure 2:**
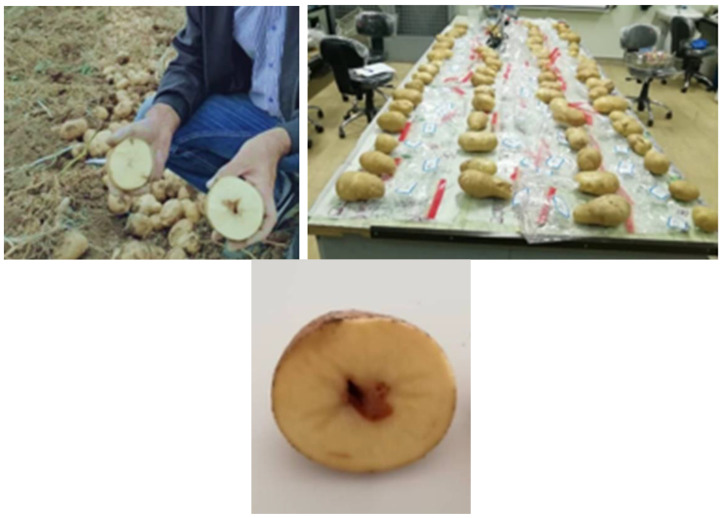
Cultivation and preparation of potato samples to classify them in healthy/defective classes.

In this study, 285 potatoes (*Solanum tuberosum* L. var Banba) were collected from Shahin Dej, Iran (location 36°40′04″ N, 46°34′01″ E), as depicted in Figure 2. All the samples were washed with water to remove earth crusts and dirt on the outside, but without removing the skin. Then, they were labeled and transferred to a laboratory for acquiring spectral information.

After spectroscopic analysis and storage of the spectral data, all the samples were cut to examine their internal state and possible damage (which, in all cases, was not recognizable by the external appearance of the tubers). This was done by human experts, since the defects were evident after cutting the samples, as shown in Figure 2. The most common diseases observed in the potatoes were brown rot and hallow heart. Out of the 285 samples, 120 healthy and 120 defective samples were selected for further analysis. The remaining 45 samples were healthy tubers which were discarded for having balanced classes.

### 2.2. Configuration of the Hardware System to Obtain Spectral Information

**Figure 3:**
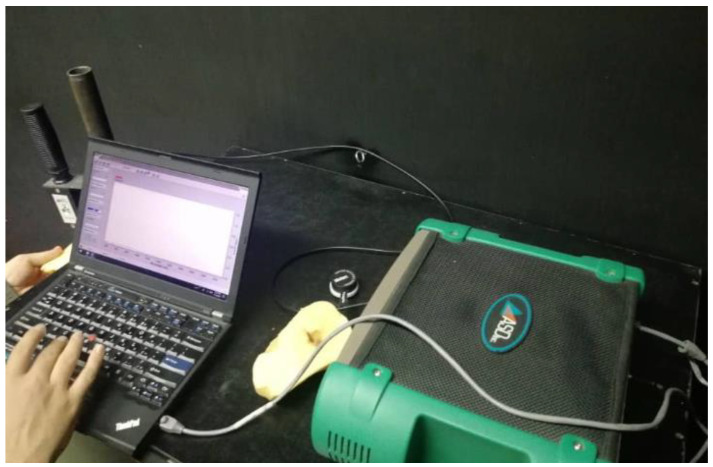
Hardware configuration for obtaining spectral data of the potato samples.

The hardware system used to capture the spectral information includes: (a) a spectrometer ASD FieldSpec 3 (Malvern Panalytical Ltd., Malvern, UK) at Vis/NIR (350–1100 nm) and short-wavelength infrared (SWIR) (1100–2500 nm); (b) three separate detectors for ranges of 350–1000, 1000–1830, and 1830–2500 nm, and (c) a laptop for data transmission and analysis. This equipment is shown in Figure 3.

The reflection mode was used to measure the spectral data [24]. In this mode, the samples are illuminated with a light of the corresponding wavelengths. Then, the spectrometer measures the spectrum of the light reflected by the sample, i.e., reflected from the potato samples skin. In this process, the projected light cannot penetrate deep into the sample, no more than 2–3 mm. The objective is not to observe the defects in a direct way but to analyze how the internal defects affect the external spectrum of the tubers at different wavelength values.

### 2.3. Preprocessing of the Obtained Spectral Information

As is well known, spectral data must be preprocessed before using it. First, using Equation (1), the reflectance was converted to absorption spectra to reduce the impact of noise due to ambient light, spectroscopy type, and others on the real data.

*Absorption spectra* = log(1/*Reflectance spectra*) (1)

**Figure 4:**
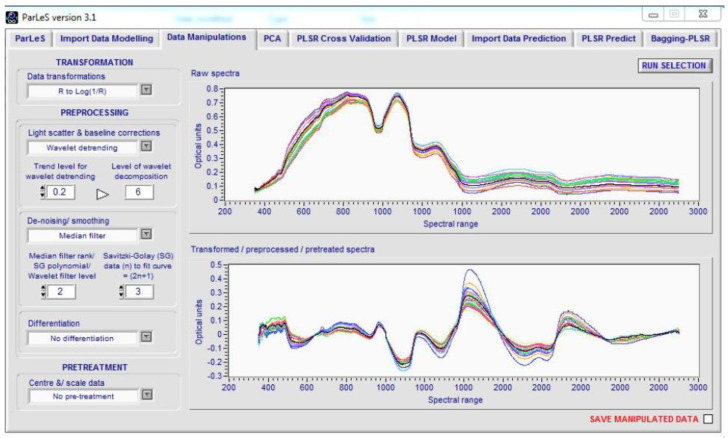
Sample application of ParLeS software (version 3.1) to preprocess the obtained spectral data for different potato samples.

Then, light scattering was corrected by the wavelength detrending algorithm. Finally, the smoothing operation was performed by the median filter using ParLeS [25], a chemical software used for multivariate modeling and forecasting (see Figure 4).

**Figure 5:**
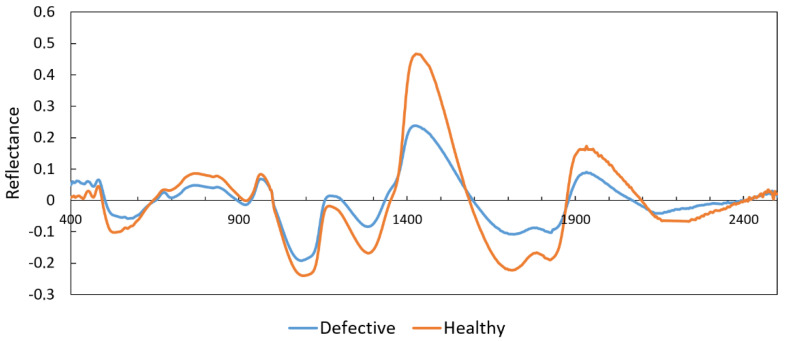
Two sample spectra of healthy and internal-defective potatoes at 400–2500 nm.

Figure 5 presents an example of a spectral plot for a healthy and a defective potato at the range 350–2500 nm. As can be seen, at the SWIR region the difference between the two samples is greater than at Vis/NIR, presenting higher peaks for the healthy sample.

### 2.4. Determination of the Optimal Wavelengths Using Hybrid ANN-CA

The ultimate goal of identifying healthy and internally defective potatoes is to develop a new detection algorithm for online grading lines. The cost and speed of operation of the portable system used in the experiments was considerable. Therefore, it would be desirable that the number of wavelengths for extracting the spectral data were reduced. For this reason, the first step of the research is to find the optimal set of wavelengths for the problem of interest. This idea of selecting a reduced number of effective wavelengths is not new, but has been successfully applied by other authors [26,27,28].

In this study, a hybrid method consisting of artificial neural networks (ANN) and the cultural algorithm (CA) was used to select the most effective wavelengths for potato classification. CA is an evolutionary algorithm that has been developed by simulating the principles of the culture of human societies. The cultural optimization process consists of two populations and belief spaces that are interconnected by acceptance functions. The population space is the same as the population of other evolutionary algorithms, which consists of a number of answers to the optimization problem. In belief space, information about population space such as status and normative information is recorded, which is used during the optimization process to determine the direction of the search in the problem-solving space.

In the hybrid method of ANN-CA, the CA heuristic sends different vectors of spectral data (selected wavelengths) as input to the ANN; the ANN is a classifier network whose output is the potato class, either healthy or defective. The ANN performs a complete train/testing process with the selected spectral data, and the results are recorded in the form of the mean squared error over the test set. The vector of wavelengths with the least mean square error is considered as the optimal vector, and the associated wavelengths are considered as the most effective wavelengths for the problem under study. Table 1 shows the structure of the hidden layers of the ANN used in the experiments.

### 2.5. Classification of Potatoes Using the Majority Voting Method

The proposed classification method is based on an ensemble of four basic classifiers combined with the majority voting (MV) rule. First, potatoes were classified using different techniques, including hybrid ANN and imperialism competitive algorithm (ANN-ICA), ANN and harmony search algorithm (ANN-HS), k-nearest neighbors analysis (KNN) and linear discrimination analysis (LDA). Then, their outputs are combined with the majority voting method. If three classifiers agree on one class, the result will be that class. This process was done using MATLAB (MathWorks, Natick, Massachusetts, USA) with the Statistics and Machine Learning Toolbox.

#### 2.5.1. Classification Using ANN-ICA

As in ANN-CA, the imperialist competitive algorithm is also an evolutionary algorithm, in this case inspired by humans and human communities, and seeks the general optimal point to solve an optimization problem [29]. By mathematically modeling the process of socio-political evolution, this algorithm provides a method for solving optimization mathematical problems with a number of random populations, each of which is called a country. Some of the best elements of the population (the elites) are selected as colonizers. The rest of the population is also considered a colony.

Depending on their power, the colonizers draw these colonies towards themselves with a certain process. The total power of any empire depends on both its constituent parts, namely the colonial state (as the central nucleus), and its colonies. With the formation of early empires, colonial rivalry between them begins. Any empire that fails to succeed in colonial competition and increase its power, will be removed from the scene of colonial competition. Thus, the survival of an empire will depend on its ability to attract the colonies of rival empires. As a result, the power of larger empires will gradually increase, and weaker empires will be eliminated.

Again, this metaheuristic process works in conjunction with an ANN. The parameters of the ANN are selected as a vector by ICA, and transferred to the ANN. The performance of the network after each proposed structure is recorded by the algorithm using the mean squared error of the classification after the train/testing of the ANN. The input of the ANN is the spectral data, and the output is the corresponding class of potato. Finally, the structure with least mean squared error is selected as the optimal structure of the ANN.

The number of hidden layers selectable by ICA was a minimum of 1 and a maximum of 3. The number of selectable neurons per hidden layer was between 1 and 25. The transfer function of each layer was selected from 13 different transfer functions, such as the tangential sigmoid. The back-propagation network training function was selected from 19 different functions. Finally, the back-propagation weight/bias learning function can be selected from 15 different functions.

After the parameters of the ANN were optimally adjusted in this process, 200 iterations were executed to evaluate the validity of the classifier. For each iteration, 60% of the samples were randomly selected for training, 30% for testing, and 10% for validation of the ANN; all of them are disjoint sets.

#### 2.5.2. Classification Using ANN-HS

This hybrid method follows the same architecture as in the previous case. A metaheuristic process, in this case the harmony search (HS) algorithm, works in conjunction with an ANN, with the purpose of selecting the optimal structure of the hyperparameters of the ANN. The difference resides in the procedure that guides the evolution of the population in the space of solutions.

HS algorithm is inspired by the modeling and simulation of the process that a composer goes through to harmonize a piece of music. The step of each musical instrument determines the beauty of the song, so the step of each instrument must be in optimal condition. Thus, the value of the objective function is determined by the values of the problem variables [30]. As a result, the architecture of the ANN selected by this method can be different to that obtained with ANN-ICA.

#### 2.5.3. Classification Using KNN

KNN is a well-known algorithm that is often used for classification problems. Basically, it consists of the following steps given a training set **T**, a new sample *s*, and a value of *k* parameter:

1. For each training sample in **T**, calculate the distance from *s* to the training sample. In our case, Euclidean distance is used as the measuring distance, which is the most common method.
2. Sort the computed distances in ascending order.
3. Select the *k* nearest training samples.
4. The output class (healthy/defective) is the class with the most samples in the previous selection.

#### 2.5.4. Classification Using LDA

Linear discrimination analysis is another common classification method in machine learning, which consists of selecting the hyperplane that best separates the existing classes. The method of LDA can be performed in three different ways: direct, hierarchical, and step-by-step. The step-by-step method is more widely used by researchers because it incorporates independent variables based on their predictive power. Therefore, in this study, the stepwise method was used [31].

### 2.6. Assessment of the Performance of the Classifiers

The performance of the classifiers was evaluated with different criteria, among the most commonly used in binary classification tasks. These criteria are recall, accuracy, specificity, precision and F-score, and graphical criteria derived from the receiver operation curve (ROC) as well as the area under the ROC curve [32]. Among these criteria, the most valuable is the accuracy, or correct classification rate (CCR), which represents the percentage of test samples that are classified correctly.

## 3. Results and Discussion

### 3.1. Optimal Wavelengths for Classifying Healthy/Defective Potatoes

As presented in Section 2, the selection of the most effective wavelengths was carried out using ANN-CA. The spectrum was divided into Vis/NIR (350–1100 nm) and SWIR (1100–2500 nm) regions. The ANN-CA process was configured to test different selections of three wavelengths, which evolved according to the CA strategy. This process was repeated until reaching convergence. Finally, the three most effective wavelengths of Vis/NIR region were 861, 883, and 998 nm, and the most effective at the SWIR region were 1539, 1858, and 1896 nm. These values correspond to some of the peaks depicted in Figure 5, where the differences between healthy and defective samples are higher.

### 3.2. Performance of the MV Classifier

Table 2 presents the performance of the MV classifier using the confusion matrix, the total CCR, and the classification error per class after 200 iterations of the experiment.

A total of 852 out of the 14,400 samples were incorrectly classified using SWIR, and 2395 using Vis/NIR. These number of incorrectly classified samples resulted in CCRs of 94.1% and 83.4%, respectively. This indicated that SWIR information was clearly more useful to detect the internal defects of the potatoes, obtaining a high accuracy that could be adequate for practical uses, while the high error using Vis/NIR could make it unpractical. It can also be observed that in both cases, the technique tended to produce an over-classification in the healthy class. For this reason, the errors in the defective class were higher than those in the healthy one. The sensitivity of the classifiers should be adjusted to produce a more balanced accuracy in the classes, if necessary.

Table 3 shows the five performance criteria of the MV classifier computed from the confusion matrix for the 200 iterations. The first evident fact is that the classification in the SWIR range was able to achieve better results than those in Vis/NIR. The accuracy in SWIR was more than 10% better, which was a considerable value. The best accuracy of 94% was feasible for practical use, while the 83% of Vis/NIR could be unpractical. Both cases tend to over-classify the samples in the healthy class, so the classification error is higher for the defective class (about 50% higher).

In both spectral ranges, the accuracy of the two classes was very similar, and therefore the results of both classes were closer to the actual value of the same class. But the precision of the healthy class was greater than the defective one in SWIR and Vis/NIR, indicating the classifier’s ability to correctly identify defective potatoes. In other words, it reveals how many of the defective samples were correctly detected (positive test result). On the other hand, the specificity of the healthy class was greater than that of the defective class, which indicated the ability of the classifier to recognize the healthy class. These results were also derived from the trend of the methods to over-classify in the healthy class.

**Figure 6:**
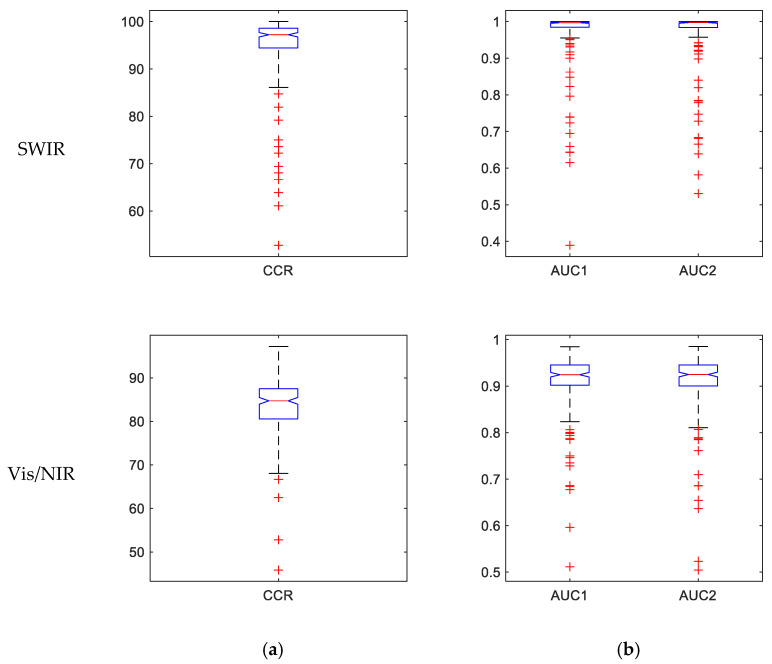
Boxplots of the performance of the MV classifier for potato classification for the 200 repetitions. (**a**) Correct classification rates. (**b**) Areas under the ROC curve (AUC), AUC1 refers to the defective class, and AUC2 to the healthy class. Upper row: classification using SWIR spectral range. Lower row: classification using Vis/NIR spectral range.

Figure 6 represents the performance of the ensemble classifier using boxplots of CCR and the areas under the ROC curve (AUC) for the 200 iterations. The compactness of these boxplots indicates the high stability of the classifiers, especially in SWIR.

**Figure 7:**
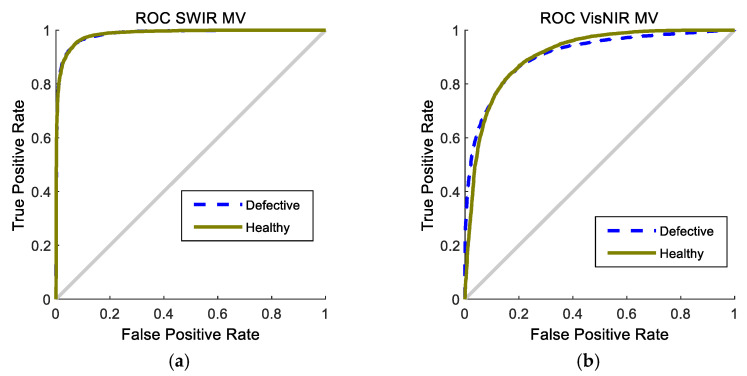
ROC curves of the MV classifier to classify potatoes in 200 iterations. (**a**) Using SWIR spectral range. (**b**) Using Vis/NIR spectral range.

Figure 7 represents the ROC curve of the ensemble classifier for the 200 iterations. As it is clear, the graphs of both classes are far from the bisector line and closer to the vertical, indicating the high performance of the classifier in the corresponding class.

Again, it can be observed that the classification in SWIR is more precise than in Vis/NIR, presenting the former a sharper curve, nearer the ideal curve. Both ROC curves in SWIR are very similar. Thus, the classifier can be conveniently adjusted to produce similar values of the false positives and false negatives. In this way, the over-classification in the healthy class could be avoided.

The average and standard deviation of the CCR and AUCs of the 200 iterations are contained in Table 4. This information can be related with the boxplots in Figure 6, indicating a good stability of the proposed classifiers in both ranges. There are only several iterations that fall far from the typical values, as shown in the red crosses in Figure 6.

### 3.3. Comparison of Different Classifiers Used for Majority Voting

Since the proposed method is a combination of four basic classifiers, it is also interesting to analyze the individual effectivity of these constituent methods. The confusion matrices, classification errors by class, and accuracies obtained by these four methods for the 200 iterations are given in Table 5, while Table 6 contains the performance criteria derived from the confusion matrices. The results obtained by the different methods are very varied, from a CCR of 70.8% for KNN using Vis/NIR, to 97.7% for LDA using SWIR.

It is notable that the maximum accuracy may not be achieved using the majority voting method, but a reliable result can be achieved using only the LDA classifier. This fact can be due to the reduction of dimensionality of the original problem after the selection of the three most effective wavelengths in SWIR and Vis/NIR. In this way, although the classes could not be linearly separable with the full spectral data, they become separable after reducing the problem to a low dimensionality. LDA is not only better than the MV method in SWIR, but also in Vis/NIR.

**Figure 8:**
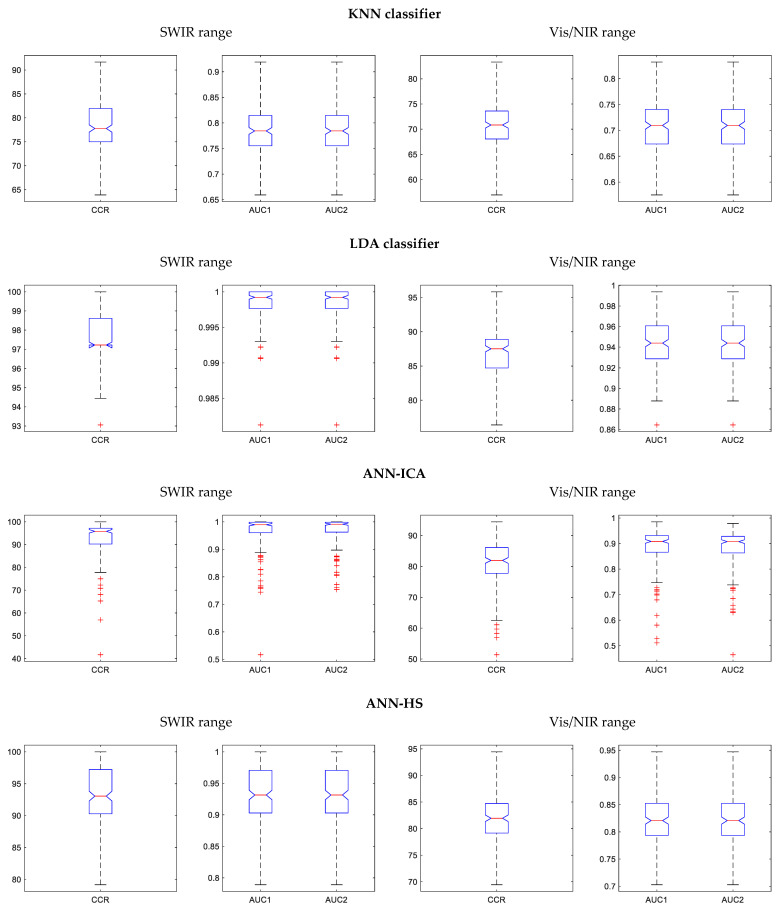
Boxplots of the performance of the four basic potato classifiers after the 200 repetitions, using the CCR and the AUC, over the two spectral ranges under consideration, SWIR and Vis/NIR.

**Figure 9:**
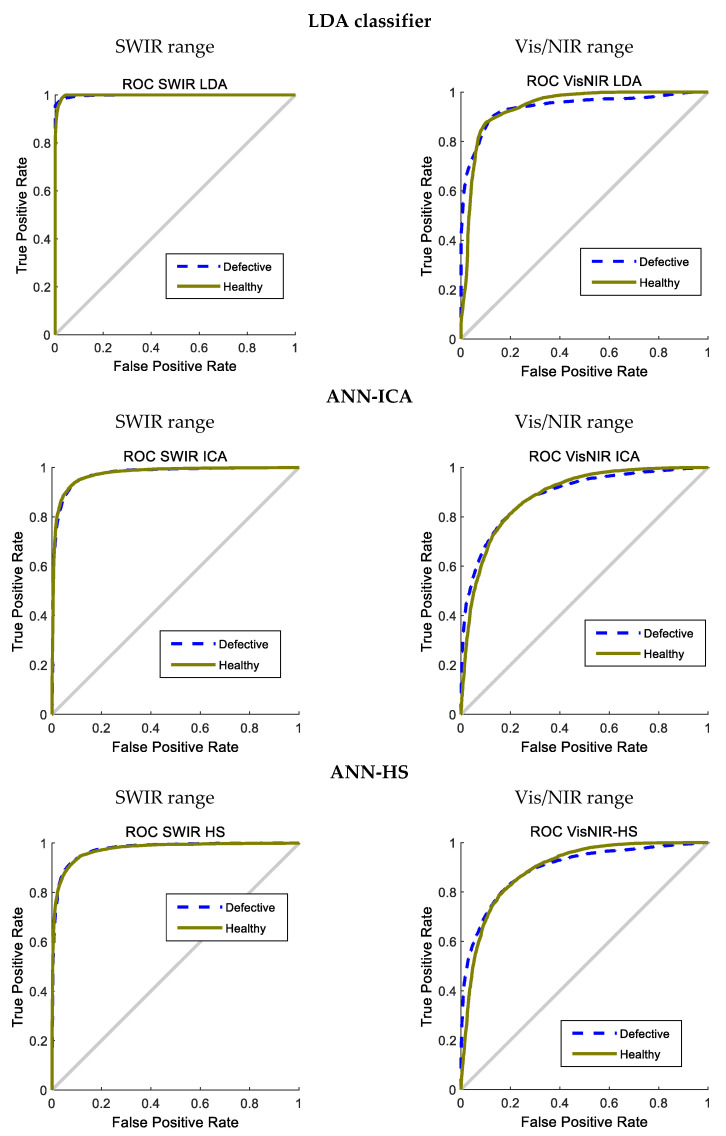
ROC curves of the four basic classifiers to classify potatoes in 200 iterations, using SWIR and Vis/NIR spectral ranges.

On the other hand, the two hybrid methods based on ANN present very similar results, with accuracies near 93%; the differences are not significant. It is also interesting to observe that SWIR consistently offers better results than Vis/NIR. This insists on the idea that the internal defects are not apparent in the visible range, but more spectral information is required. In fact, the three wavelengths selected in Vis/NIR (861, 883, and 998 nm) correspond to the NIR range. More information on the results of the basic classifiers is presented in Figure 8 and Figure 9, containing boxplots and ROC curves after the 200 iterations.

Since the KNN classifier only has a working mode and cannot be configured to be more tolerant or restrictive (unlike the other methods), its ROC curve only has one point. For this reason, the ROC curve of KNN is not included in Figure 9. Again, the superiority of LDA over the rest of the methods is evident, although it is not able to achieve good results in Vis/NIR.

### 3.4. Comparison of Either Using the Whole Spectral Range or the Selected Wavelengths

As discussed above, to develop an inexpensive capture device and a high-speed classifier, it is necessary to identify the most effective wavelengths for the problem of study. This would allow the development of simplified spectral cameras that capture only a specific range of the spectrum, and the latter analysis in a portable device. For this reason, it is interesting to analyze the difference between using all the available spectral information or using only the selected wavelengths. So, the ensemble classifier was applied on the same dataset of potatoes using all the spectral information of the samples, with the same constituent methods and partition of the samples in train/test/validation disjoint sets.

The results of the classification using all the wavelengths and the most effective ones are compared in Table 7.

These results show that, although the detection rate obtained by using all the spectral information is slightly higher, the standard deviation produced is also large. In fact, the difference in the best case (using SWIR) is only about 2%, much lower than the standard deviations of both methods (above 7%). To study the statistical significance of this fact in more detail, a two-tailed t-test method was used to analyze the given differences. The null hypothesis (H_0_) is that the mean CCRs obtained using all the spectral data and using only the most effective wavelengths are equal, and the alternative hypothesis (H_a_) is that they are different. The results of the test are presented in Table 8.

The test shows that there is no statistically significant difference between the CCRs obtained for these two datasets. In consequence, the option of using only the three selected wavelengths in SWIR is justified. It has been considered unnecessary to compare the CCRs of SWIR and Vis/NIR, since the differences in this case are clearly larger than the observed standard deviations.

Finally, the obtained results have been compared with other works in the literature that are similar to our study, although all of them use their own datasets. These works are specialized on certain potato diseases. This is the case in the study proposed by Liang et al. [33], focused on the detection of zebra chip disease in potatoes using spectral information. They analyzed Vis/NIR/IR, observing that the wavelengths in the visible range were the most effective (468, 582, 680, and 720 nm), achieving an accuracy of 97%. This accuracy was similar to our findings using LDA, although in our case the Vis range was not found to be effective. This supports the idea that different diseases could have spectral signatures in different parts of the spectrum. Additional multi-disease experiments in potatoes would be required to validate this hypothesis.

Another closely related work is the method proposed by Zhou et al. [18], where the detection of blackheart disease in potatoes was studied using spectroscopy and machine learning. They observed that the most effective values were located in the Vis/NIR range, between 678 and 839 nm, selecting six optimal wavelengths. The authors centered their attention on the morphological corrections, for example, with respect to the height of the tubers. The accuracy obtained ranged between 96.53% and 97.11%, which is also very close to the 97.7% in our best method. In any case, as previously stated, the results are not directly comparable since our work is not specific to a single disease and, consequently, the datasets are different. Even so, it is very interesting to observe that all the state-of-the-art methods are able to obtain high accuracies slightly above 97%.

## 4. Conclusions

The purpose of this research was to accurately detect potatoes with internal defects, such as brown rot, hollow heart, and black heart, using simple external spectroscopy analysis. The study was conducted at the regions of Vis/NIR (350–1100 nm) and SWIR (1100–2500 nm) of the spectrum. First, the most effective wavelengths were determined for each region using a hybrid ANN-CA approach. Then, an ensemble classifier was proposed using the majority voting rule on four constituent methods: ANN-ICA, ANN-HS, LDA, and KNN. The results of the ensemble classifier showed that the correct classification rate of the samples was 94.1% for the SWIR range, and 83.4% for the Vis/NIR range. SWIR region was found to be the most appropriate for detecting internal potato defects. Interestingly, one of the constituent methods, LDA, was able to achieve the best classification results, with 97.7% accuracy in SWIR. This method would be preferable over the ensemble classifier, due to its precision and simplicity. The effectivity of using only the three best wavelengths was also compared with respect to the use of the whole spectrum. In this case, it was observed that there were not statistically significant differences between both methods, so obviously, the best option would be to use only the selected wavelengths.

These findings will help to develop new capture devices that could be more simplified and practical to use, since only the wavelengths of interest would be captured. With the development of simplified portable devices, the detection technology could be applied either in the field or in factories at their handling processes. In the field, it can be used to perform random sampling of the tubers to carry out an early detection of possible plant diseases. Inside the factory, it can be used in a processing line for quality inspection, where the defective potatoes would be discarded. Both the capture of just three wavelengths and classification times using LDA allows potential real-time processing in practice. Another future line of research is to analyze how the different diseases affect potato spectral signatures at various wavelengths. Further experiments would be needed to classify the defective samples according to the types of diseases observed.

## Figures and Tables

**Table 1:** Structure of the ANN used to select effective wavelengths in the ANN-CA process.

Number of Hidden Layers	Number of Neurons Per Layer	Transfer Function	Backpropagation Function	Backpropagation Weight/Bias Function
2	1st: 12, 2nd: 16	poslin	logsig, purelin	trainbfg

**Table 2:** Evaluation of the performance of the MV classifier using the confusion matrix, the error rate per class, and the CCR, after 200 train/test iterations.

Spectral Range	Class	Healthy	Defective	Total Data	Misclassified (%)	CCR
SWIR	Healthy	6877	337	7214	4.7	94.1
	Defective	515	6671	7186	7.2	
Vis/NIR	Healthy	6303	1004	7307	13.7	83.4
	Defective	1391	5702	7093	19.6	

**Table 3:** Performance criteria of the MV classifier after 200 iterations in the two spectral ranges.

Spectral Range	Class	Recall (%)	Accuracy (%)	Specificity (%)	Precision (%)	F-Score (%)
SWIR	Defective	95.2	94.1	93.0	92.8	94.0
	Healthy	93.0	94.1	95.2	95.3	94.2
Vis/NIR	Defective	85.0	83.4	81.9	80.4	82.6
	Healthy	81.9	83.3	85.0	86.3	84.0

**Table 4:** Mean and standard deviation (SD) of the MV classifier for the 200 iterations, using the CCR and the AUCs (AUC_1_ stands for the defective class, and AUC_2_ for the healthy class), over the two spectral ranges.

Spectral Range	Value	CCR	AUC_1_	AUC_2_
SWIR	Mean	94.1	0.980	0.980
	SD	8.04	0.065	0.067
Vis/NIR	Mean	83.4	0.900	0.900
	SD	6.76	0.065	0.068

**Table 5:** Evaluation of the performance of the four basic classifiers using the confusion matrix, the error rate per class, and the CCR after 200 train/test iterations.

Basic Method	Spectral Range	Class	Healthy	Defective	Total Data	Misclassified (%)	CCR (%)
KNN	SWIR	Healthy	6010	1204	7214	20.0	78.4
		Defective	1911	5275	7186	36.2	
	Vis/NIR	Healthy	5424	1883	7307	34.5	70.8
		Defective	2329	4764	7093	48.9	
LDA	SWIR	Healthy	7214	0	7214	0	97.7
		Defective	338	6848	7186	4.9	
	Vis/NIR	Healthy	6698	609	7307	9.1	87.1
		Defective	1245	5848	7093	21.3	
ANN-ICA	SWIR	Healthy	6720	494	7214	7.4	92.6
		Defective	573	6613	7186	8.7	
	Vis/NIR	Healthy	6150	1157	7307	18.8	81.1
		Defective	1571	5522	7093	28.4	
ANN-HS	SWIR	Healthy	6841	373	7214	5.5	93.0
		Defective	636	6550	7186	9.7	
	Vis/NIR	Healthy	6275	1032	7307	16.4	82.2
		Defective	1525	5568	7093	27.4	

**Table 6:** Different criteria for evaluating the performance of different classifiers to classify potatoes in 200 iterations.

Basic Method	Spectral Range	Class	Recall (%)	Accuracy (%)	Specificity (%)	Precision (%)	F-Score (%)
KNN	SWIR	Defective	81.4	78.4	75.9	73.4	77.2
		Healthy	75.9	78.4	81.4	83.3	79.4
	Vis/NIR	Defective	71. 7	70.8	70.0	67.2	69.3
		Healthy	70.0	70.8	71.7	74.3	72.0
LDA	SWIR	Defective	100	97.7	95.5	95.3	97.6
		Healthy	95.5	97.7	100	100	97.7
	Vis/NIR	Defective	90.6	87.1	84.3	82.4	86.3
		Healthy	84.3	87.1	90.6	91.7	87.8
ANN-ICA	SWIR	Defective	93.0	92.6	92.1	92.0	92.5
		Healthy	92.1	92.6	93.0	93.6	92.6
	Vis/NIR	Defective	82.7	81.1	79.7	77.9	80.2
		Healthy	79.7	81.1	82.7	84.2	81.8
ANN-HS	SWIR	Defective	94.6	93.0	91.5	91.1	92.8
		Healthy	91.5	93.0	94.6	94.8	93.1
	Vis/NIR	Defective	84.4	82.3	80.4	78.5	81.3
		Healthy	80.4	82.3	84.4	85.9	83.1

**Table 7:** Comparison of mean and standard deviation (SD) values of CCR and AUCs of the ensemble classifier using either all the spectral data or using only the selected wavelengths, over SWIR and Vis/NIR spectral ranges.

Wavelengths Used	Spectral Range	Value	CCR	AUC_1_	AUC_2_
Effective	SWIR	Mean	94.1	0.98	0.98
wavelengths		SD	8.04	0.065	0.067
Effective	Vis/NIR	Mean	83.4	0.90	0.90
wavelengths		SD	6.76	0.065	0.068
Full spectra	SWIR	Mean	96.3	0.98	0.98
		SD	7.49	0.065	0.067
Full spectra	Vis/NIR	Mean	86.1	0.903	0.902
		SD	15.6	0.147	0.150

**Table 8:** T-test of the difference of the total correct classification rate (CCR) of the MV classifier using all the spectral data and using only the most effective wavelengths in SWIR.

		Mean	Standard Deviation	T-Value	Degree of Freedom	Significance
Pair 1	Effective-Whole	−2.475	0.332	−10.53	1	0.060

## Data Availability

The data presented in this study are available on request from the corresponding author. The data are not publicly available since they are partially owned by the laboratory where the analyzes were carried out.
